# Transforming Public Health Practice with Artificial Intelligence: A Framework-Driven Approach

**DOI:** 10.3390/healthcare14030385

**Published:** 2026-02-03

**Authors:** Obinna O. Oleribe, Florida Uzoaru, Adati Tarfa, Olabiyi H. Olaniran, Simon D. Taylor-Robinson

**Affiliations:** 1 Department of Health Sciences, School of Public Health and Health Sciences, California State University, Dominguez Hills, 1000 E Victoria Street, Carson, CA 90747, USA; 2 Center for Family Health Initiative (CFHI), 286 W Sparkleberry Avenue, Orange, CA 92865, USA; 3 College of Nursing and Health Sciences, Southeastern Louisiana University, Hammond, LA 70402, USA; florida.uzoaru@selu.edu; 4 Department of Internal Medicine, Yale School of Medicine, New Haven, CT 06511, USA; adati.tarfa@yale.edu; 5 Division of Vascular & Endovascular Surgery, Beth Israel Deaconess Medical Center, Harvard Medical School, Boston, MA 02215, USA; oolanira@bidmc.harvard.edu; 6 Department of Surgery and Cancer, St Mary’s Hospital Campus, Imperial College London, London W2 1NY, UK; s.taylor-robinson@imperial.ac.uk

**Keywords:** artificial intelligence, public health practice, policy development, health equity, ethics, bias and fairness, AI transparency, regulatory frameworks, public health leadership, human–AI collaboration

## Abstract

Background: The emergence of artificial intelligence (AI) has triggered a global transformation, with the healthcare sector experiencing significant disruption and innovation. In current public health practice, AI is being deployed to power various aspects of public functions, including the assessment and monitoring of health, surveillance and disease control, health promotion and education, policy development and planning, health protection and regulation, prevention services, workforce development, community engagement and partnerships, emergency preparedness and response, and evaluation and research. Nevertheless, its use in leadership and management, such as in change management, process development and integration, problem solving, and decision-making, is still evolving. Aim: This study proposes the adoption of the **Public Health AI Framework** to ensure that inclusive data are used in AI development, the right policies are deployed, and appropriate partnerships are developed, with human-relevant resources trained to maximize AI potential. Implications: AI holds immense potential to reshape public health by enabling personalized interventions, democratizing access to actionable data, supporting rapid and effective crisis response, advancing equity in health outcomes, promoting ethical and participatory public health practices, and strengthening environmental health and climate resilience. Achieving this goal will require a deliberate and proactive leadership vision, where public health leaders move beyond passive adoption to collaborate with AI specialists to co-create, co-design, co-develop, and co-deploy tools and resources tailored to the unique needs of public health practice. Call to action: Public health professionals can co-innovate in shaping AI evolution to ensure equitable, ethical, and value-based public health.

## 1. Introduction

The global public health landscape is undergoing a profound transformation, fueled by the emergence, expansion, and rapid adoption of artificial intelligence (AI). AI is not a new concept, as scientists started asking the question “Can machines think?” as far back as World War II (1942–1945), which culminated in the coining of the word in the Dartmouth Conference in 1956 by John McCarthy [1,2,3]. However, since 2022, AI technologies have introduced innovative solutions to long-standing public health challenges. While digital health tools have been in use for decades, AI stands apart due to its unique ability to synthesize information, generate human-like responses, and personalize interventions at scale [2,3,4,5].

While there is no universally accepted definition, AI is broadly defined as the capacity of machines to perform tasks that would typically require human intelligence [6]. This paper defines the scope of AI to include a range of interrelated technologies that underpin its functionality and public health applications. These include machine learning (ML), where algorithms learn patterns from data for classification, prediction, and decision support [7]; deep learning (DL), a subset of ML involving multilayered neural networks used for complex pattern recognition in imaging, speech, and natural language [8]; and natural language processing (NLP), which allows machines to understand, interpret, and generate human language in contexts such as health communication and epidemiological reporting [9]. This scope also encompasses explainable AI (XAI), which enhances transparency and interpretability in model outputs [10]; computer vision, used to extract meaningful insights from visual inputs like radiographs or satellite imagery [11]; cognitive computing, which mimics human reasoning by combining AI, ML, and NLP [12]; and generative AI (GenAI), a fast-growing field that creates new data or content by learning patterns from existing datasets [13].

This definitional framework provides a basis for examining AI’s growing influence in public health practice. It also supports the case for deliberate, proactive leadership to guide its ethical, equitable, and population-centered integration [14,15]. However, as the utilization of AI in public health continues to expand across systems, functions, and jurisdictions, a new question emerges: how can public health institutions guide the responsible adoption of AI in alignment with leadership, governance, workforce capacity, and equity across the AI lifecycle?

This manuscript addresses this question to inform practice as AI adoption in public health accelerates, and is presented as a perspective and conceptual framework paper. It examines the role of artificial intelligence across essential public health functions, identifies key implementation challenges, and proposes a Public Health AI Framework to support ethical, equitable, and population-centered integration of AI within public health systems. The conceptual Public Health AI Framework specifically addresses the organizational structures, governance mechanisms, and implementation contexts unique to governmental public health systems, a gap not fully addressed by existing AI governance frameworks. To situate this contribution, this paper examines the evolution of public health systems, investigates how AI is being used across essential public health functions, identifies critical barriers to implementation, and proposes a framework to guide the responsible integration of AI in public health systems. To understand AI’s current role in public health, it is important to first examine the evolution of public health systems and practices. The following section outlines the evolution of public health and the conceptual roadmap guiding the manuscript, and describes the structure through which these arguments are developed.

## 2. Evolution of Public Health

Since the 19th century, public health practice has evolved through different phases, each reflecting a shift in scientific knowledge, societal priorities, and institutional structures. Public Health 1.0 focused on establishing fundamental infrastructure, including safe water, sewage systems, waste management, food safety, and housing reforms, alongside the development of governmental public health agencies to institutionalize these functions. Public Health 2.0, which spanned from the mid-twentieth century to the early 2000s, consolidated modern public health practice as the scope of public health expanded to address chronic diseases, maternal and child health, vaccination, health education, environmental health [16,17], and the rise in public health interventions, including infectious disease surveillance and control [18,19,20].

Public Health 3.0 introduced a bold new framework for modern public health, first articulated by the U.S. Department of Health and Human Services in 2016 [21]. It repositioned government agencies to convene and lead partnerships across housing, transportation, education, environmental sectors, and business, resulting in governmental commitments to health equity, data modernization, and various structural changes through appropriate policy formulation, system improvement, and environmental interventions [21,22,23]. At its core, this model prioritizes digital innovation and mental health integration, recognizing the urgent need for adaptive leadership to counter burnout, demographic shifts, and vulnerabilities exposed during crises such as COVID-19 [24,25,26,27]. At the same time, the rise of big data and AI provided powerful tools for evidence-based action but required ethical governance to safeguard equity [28,29]. Thus, Public Health 3.0 repositioned public health as a collaborative enterprise rooted in equity and community leadership by uniting communities, modernizing systems, and embedding mental health as a central priority. It stands as a defining call to action and a blueprint for resilient, equitable health systems capable of addressing today’s challenges while preparing for the crises of tomorrow [21,30].

Public Health 4.0 emerged in the 2020s primarily to integrate digital technologies, AI, and precision public health approaches into routine practice [31,32,33]. Central to this digital transformation is the integration of AI, which is reshaping how public health functions are operationalized. AI can now be applied across those interventions that were developed across the lifespan of public health, from infectious disease control to addressing social determinants, health equity, ethics, and community engagement to surveillance and prevention to workforce development and emergency response. The parallel evolution of AI technologies and public health systems underscores the need for deliberate, coordinated approaches to AI integration grounded in public health values. While AI offers substantial potential to enhance decision-making and population health impact, its benefits depend on leadership, governance, workforce capacity, partnerships, and ethical oversight.

## 3. Application of AI in Public Health Practice

AI adoption is playing a significant role in Public Health 4.0, and is applicable in all aspects of public health, from health education and communication, surveillance and epidemiology, and data science and big data management, to evaluation and research (Table 1).

AI is used as chatbots and virtual assistants to translate complex medical terminology into plain language. They answer questions in real time and provide tailored health messaging in multiple languages [38,50]. During the COVID-19 pandemic, local health departments piloted AI chatbots to answer public inquiries about testing, vaccines, and isolation protocols to reduce call center burdens and provided 24/7 assistance in the United States [51,52]. These AI engagements helped improve public health reach, quality of care, and reduce morbidity and mortality rates. With increasing global diversity, the need for advanced versions of these tools and resources cannot be overemphasized. When used appropriately and responsibly, AI-powered tools help learners develop creative thinking and build decision-making skills through real-world scenarios. Furthermore, AI is used extensively in epidemiology to generate synthetic datasets for disease surveillance and automate the writing of epidemiological reports, thereby reducing the workload on frontline epidemiologists. AI has also been used to simulate disease outbreaks [53,54]. The South Korean Centers for Disease Control incorporated AI into their flu and COVID-19 tracking systems to generate weekly bulletins to free up time for experts to analyze and plan appropriate responses [55]. Also, public health educators are using AI to create course content, assessments, rubrics, simulations, and interactive modules for professional training [56,57,58]. In Canada, health professional training programs are beginning to integrate AI into classroom learning. For example, the University of British Columbia’s UBC Health piloted an AI-powered chatbot, “Marie Parker,” within a simulated electronic health record system [59]. This AI-driven case study activity enables students to engage in outbreak-style investigations, policy decision-making, and ethical discussions in a controlled digital classroom environment, demonstrating the potential of AI to enrich public health and interprofessional education [59].

Evidence shows that AI is increasingly being integrated into public health and clinical care across LMICs, with a range of applications demonstrating both promise and complexity. AI-based tools have been deployed for screening infectious and non-communicable diseases, such as malaria, tuberculosis, diabetes, and chronic kidney disease, often supporting early detection and clinical decision-making [60]. In rural India, a public–private partnership deployed an AI chatbot trained in regional dialects to educate communities about maternal health and vaccine schedules, significantly improving health knowledge retention [61,62]. In imaging, AI has enhanced the interpretation of point-of-care ultrasound and chest radiographs, improving operational efficiency in resource-limited settings [63,64]. Primary care diagnostic support systems are also emerging, offering assistance in complex case triage and differential diagnosis [65]. However, the performance of AI systems can vary significantly across use cases, populations, and conditions. For example, dermatology-focused AI tools have shown limitations when applied to darker skin tones, underscoring the importance of inclusive design and training data [66,67]. In Kenya, a GenAI-powered mobile mental health virtual therapist showed promise in improving mental health outcomes among adolescents in urban slums, offering scalable and cost-effective support [68,69]. These developments reflect both the growing role of AI in LMIC health systems and the ongoing need for context-specific evaluation and adaptation. Beyond direct public health interventions, AI is increasingly instrumental in enhancing healthcare management operations as organizations leverage it to support decision-making, for predictive maintenance of medical equipment, and to optimize supply chains for vaccines and essential medicines [70,71]. In Africa, both Rwanda and Nigeria have utilized drone technology to deliver blood supplies to remote regions with limited infrastructure [72,73,74].

Despite the success recorded with AI adoption, AI has failed to achieve some set objectives in some cases. For instance, during the COVID-19 pandemic, several AI-driven forecasting models produced inconsistent or inaccurate projections of case counts, hospitalizations, and mortality due to poor data quality and reporting lags, non-stationary dynamics, overfitting to early-pandemic data, and limited integration of social and behavioral variables [75,76]. This resulted in decision-makers losing confidence, and models were sidelined, and policies defaulted to reactive measures. Similarly, Google Flu Trends significantly overestimated influenza prevalence and was eventually discontinued due to its reliance on proxy signals without proper epidemiologic grounding, algorithm drift as user behavior changed, and lack of transparency and external validation [77]. AI-enabled and digital contact tracing tools deployed globally failed to achieve projected uptake due to privacy concerns and mistrust, digital divide and smartphone access gaps, weak integration with public health workflows, and insufficient community engagement [78,79].

Some AI tools used to prioritize care or allocate resources underestimated risk in marginalized populations as the training data reflected historical inequities, or they used cost or utilization as a proxy for need, and there was an absence of equity auditing [80]. AI-based syndromic surveillance systems triggered false alerts for outbreaks that did not materialize due to noise from seasonal trends, media coverage, or behavioral changes: insufficient human oversight and poor threshold calibration [81,82]. False outputs were also seen when AI was used in prior authorization, which has wrongfully denied treatment to those who needed it, although it significantly reduced processing time, administrative load, and improved some patients’ access to necessary treatments [83,84,85]. These examples illustrate both the transformative potential and critical risks of AI in public health. Success requires not only technical capability but also governance, human oversight, and equity-centered design. Despite these advances in artificial intelligence that have continued to generate new possibilities for public health practice, associated challenges inform the framework proposed in this paper and necessitate examination of how AI capabilities are anticipated to evolve within public health practice, and prompt consideration of how emerging AI capabilities may shape future public health functions.

## 4. Emerging Opportunities for GenAI in Public Health Practice

As AI technology matures, applications are evolving from supplementary support tools to central instruments in strategic planning, population health management, and systems-level transformation. Table 2 outlines emerging and near-term AI capabilities across the 10 Essential Public Health Services, including applications currently in development and those anticipated within the next decade.

AI is expected to design highly tailored public health interventions by leveraging individual and population-level data to address specific risk profiles, behaviors, and social determinants of health. Unlike traditional approaches, AI may adapt messaging and interventions in real time to suit cultural, linguistic, and behavioral contexts, as well as helping to bridge the data gap among historically marginalized populations that have remained underrepresented in health by generating synthetic data. Such data will model underrepresented groups and translate technical information into accessible formats to democratize data insights. Also, as timely and accurate information is essential in disasters, AI can rapidly generate, translate, and disseminate localized health advisories, simulate outbreak scenarios, and support logistics planning under high uncertainty.

AI can be useful in modeling ethical dilemmas and drafting policies that incorporate diverse stakeholder viewpoints. Furthermore, models can be trained on environmental data to forecast the health impacts of heatwaves, air pollution, or climate-induced migration to generate adaptation strategies for at-risk populations. Similarly, scientists can use AI to develop more accurate GIS mapping of strategic sites and locations to generate narratives and visualizations that can help local governments plan adaptive infrastructure and health services for displaced communities.

Although the place of AI in public health practice is both promising and multidimensional, there are critical challenges that must be addressed before we can maximize AI in public health practice. The applications outlined in Table 1 and Table 2 demonstrate AI’s expanding role across public health domains. However, translating these capabilities into equitable, effective practice requires confronting several critical ethical, governance, infrastructural, workforce-related and other implementation challenges.

## 5. Critical Challenges in the Implementation of AI in Public Health

To be impactful, robust ethical frameworks and inclusive leadership must guide the adoption and deployment of AI. This requires public health leaders to incorporate relevant ethical frameworks, guidelines, and guardrails when adopting, deploying, and using AI. Policy frameworks such as the WHO comprehensive guidance to support member states’ responsible regulation of AI technologies in healthcare [99], the FUTURE-AI initiative international consensus guidelines to ensure the trustworthiness of AI in clinical practice [100], the United States Food and Drug Administration (FDA) AI/ML-Based Software as a Medical Device (SaMD) Action Plan [101], and the European Union’s Artificial Intelligence Act [102] are some of the global attempts to regulate AI technologies. When applied appropriately, these frameworks will help minimize the risk of algorithmic bias and inequity, promote responsible use, foster creative thinking, and ensure ethical practice in the use of AI.

Current historical or real-world data used in the development of AI models can reflect social, racial, gender, environmental, or geographic biases and injustices, with the resulting tools risking the perpetuation and amplification of existing disparities in healthcare delivery. For instance, a widely used model developed to predict patient health needs in U.S. hospitals was found to underestimate the health needs of Black/African American patients, resulting from biased training data that used healthcare costs as a proxy for health status [79]. To minimize such biases, it is necessary to audit models for racial and socioeconomic biases [103]. Additionally, the lack of transparency and explainability in AI outputs undermines trust, making it difficult for public health professionals to validate or interpret model recommendations. Similar distrust is observed among clinicians, who have expressed hesitation in relying on AI’s output due to a lack of insight into how it prioritizes patient urgency, needs, and diagnosis [104]. Integrating explainability features that provide a rationale for each triage decision will improve adoption.

Furthermore, AI systems require large volumes of data to function effectively. Without proper data governance structures in place, there is a significant risk of violating individual privacy and eroding public trust. Models must respect individuals’ autonomy and not fail to obtain informed consent for the data it collects, which may result in public backlash and concerns about the misuse of personal data and suspension of the program [105]. The lack of robust community engagement in the design, development, and rollout of AI tools, a key principle of public health, is another challenge, as many AI tools are designed, developed, and operationalized with minimal input from the populations they plan to serve, leading to poor contextual fit, low adoption, and avoidable harm. Authors’ lived evidence reinforces this concern, showing that when users are meaningfully engaged early in the design, technologies become more responsive, effective, and widely adopted. Similarly, Sandhaus et al. documented that the involvement of users in the design phase resulted in enhanced creativity and faster design iterations in an educational project [106]. Participatory design and redesign workshops with users will develop more localized and culturally relevant versions that will result in enhanced user satisfaction. To support these initiatives and guide responsible AI integration, we propose the following framework.

## 6. Public Health AI Framework and Leadership Imperatives

**Figure 1:**
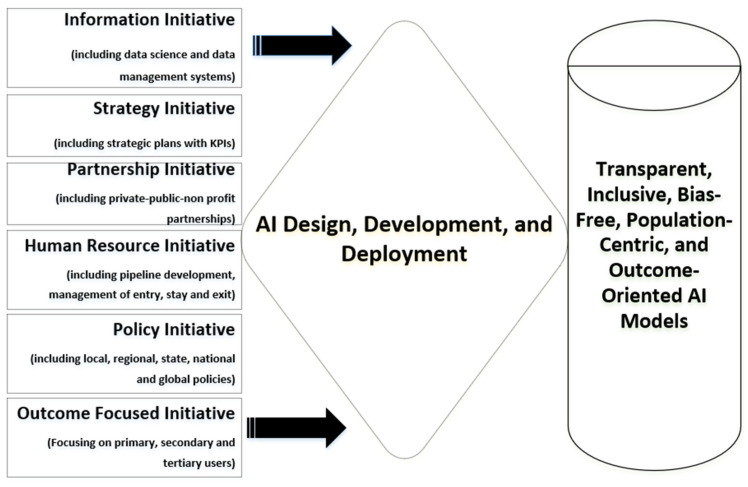
Public Health Artificial Intelligence Framework.

To minimize AI-related challenges and risks, public health leaders, policymakers, and developers must work collaboratively to develop AI tools for public health practice. The user-centric Public Health AI Framework (Figure 1) could guide this process. This conceptual framework was developed through an integrative synthesis of the existing AI governance literature, analysis of documented AI implementations in public health (as shown in Table 1 and Table 2), public health leadership models, the established artificial intelligence ethics literature, identification of gaps in current governance models that do not adequately address governmental public health contexts, and the authors’ applied implementation experience, rather than through formal consensus methods or empirical validation. Building on the implementation challenges identified in the preceding section, the proposed Public Health AI Framework supports coordinated, ethical, and outcome-oriented integration of AI within public health systems. Distinct from existing AI governance models that primarily emphasize technical or regulatory considerations, the framework adopts a user-centric and system-level orientation, foregrounding leadership stewardship, workforce readiness, cross-sector partnerships, and alignment with population health objectives. Figure 1 presents the six core components of the framework and illustrates their interrelationships within public health practice.

The proposed six-component Public Health AI Framework (Figure 1) will inform AI model development and operationalization, leading to better public health outcomes. The proposed framework will empower leaders with the ability to pre-empt challenges that may arise in AI adoption, integration, and use. Adopting the six components of the framework will move public health leaders from mere passive AI adoption to co-designers, co-creators, and developers of AI tools, as previously published [107]. These components of the framework are organized to reflect the AI lifecycle in public health practice: from foundational data infrastructure (Information) and strategic planning (Strategy), through development and implementation (Partnership, Human Resources, Policy), to continuous evaluation (Outcomes). This structure ensures systematic integration across all stages of AI adoption.

Component 1: Information Initiative: Information has always been the backbone of public health practice. In the age of big data, the capacity to collect, curate, and transform information into actionable knowledge determines the success of AI adoption. Public health professionals must first identify available datasets, map existing gaps, and evaluate their quality and representativeness. This requires collaboration across sectors—health systems, academia, technology partners, and communities—to co-create information ecosystems that are both robust and equitable. In this initiative, data are not just a resource, but the fuel for developing, training, and deploying AI models in public health. Effective data hygiene and governance, interoperability standards, and ethical safeguards to protect individual privacy, mitigate bias, and ensure inclusivity are essential to guaranteeing that information-driven AI systems advance health equity, rather than exacerbate disparities. Without deliberate investment in information architecture, public health AI will be built on fragile foundations.

Component 2: Strategy Initiative: Strategic planning is critical and will guide the responsible adoption of AI in public health. Using the Management by Objectives (MBO) paradigm, public health leaders must develop short-, medium-, and long-term strategies that articulate clear goals, measurable outcomes (with key performance indicators), and adaptive pathways for AI integration [108]. This includes defining the information needs of each stage, sequencing adoption in alignment with system readiness, and establishing a sustainable pace of change that avoids disruption while maximizing impact. A well-structured strategy initiative will ensure that AI is not introduced haphazardly but integrated deliberately within the broader public health mission. It provides direction on governance, investment, capacity building, monitoring, and evaluation, while embedding ethical and equity considerations from the outset. A strategic foresight will prevent fragmented implementation and position AI as a transformative tool for disease prevention, surveillance, and health promotion.

Component 3: Partnership Initiative: The development of effective AI tools for public health practice requires interdisciplinary collaboration. Public health specialists must partner with biomedical engineers, computer scientists, and information technology experts to co-design, co-create, and co-develop AI-driven resources. This participatory approach ensures that AI solutions are current, contextually relevant, comprehensive, inclusive, ethically grounded, and aligned with public health priorities, rather than being retrofitted products designed solely for clinical or commercial use. Instead of waiting for fully developed technologies to be handed down, public health professionals can be engaged in the innovation process from the outset—from conceptualization and design through development, deployment, and operationalization. This collaborative model will accelerate translation, improve usability, and foster systems that are more equitable and responsive to real-world public health challenges. Involving diverse voices, including community stakeholders, community-based and non-profit organizations, and service beneficiaries, will further strengthen trust and adoption. Additionally, the creation of partnerships directly addresses the ethical challenge of algorithmic bias discussed in Section 5 [79], where non-representative training data led to discriminatory underestimation of healthcare needs in Black/African American populations.

Component 4: Human Resource Initiative: Building appropriate human capital is central to the sustainable integration of AI in public health. Schools and colleges of public health can proactively and deliberately design curricula that equip future professionals with the skills necessary to adopt, assimilate, and use AI tools effectively. This training must go beyond surface-level skills such as prompt engineering and extend into the technical, ethical, and applied dimensions of AI. Competency development can span multiple domains such as data science literacy, algorithmic transparency, ethics and equity, and the integration of AI into the three core functions of public health: assessment, policy development, and assurance [17]. Continuing professional education for the existing workforce will be equally important to prevent generational divides in skillsets. By embedding AI competencies in training pipelines, public health institutions can ensure that professionals are not mere passive users but informed learners and leaders in shaping AI evolution for public and population health.

Component 5: Policy Initiative: The integration of AI into public health requires enabling policy and regulatory frameworks that safeguard equity, transparency, accountability, responsible use, and ethical practice. Several jurisdictions are already establishing precedents. The European Union (EU) AI Act has set a global benchmark, influencing member states in developing national AI health policies [102]. National strategies should explicitly designate public health as a priority sector for AI adoption. Individual countries or regional supra-national organizations, such as the African Union or the EU, should launch health chatbots that embed consent mechanisms and safeguard user rights, and use culturally safe technologies by engaging indigenous communities and digital rights advocates.

Beyond national or supra-national initiatives, global organizations like the WHO and UNESCO could monitor guidelines on the ethical and responsible use of AI in health. Public health leaders can leverage existing and future frameworks to develop comprehensive AI policies that are tailored to population health needs. Such policies must integrate provisions aligned with the 10 Essential Public Health Services, ensuring that AI supports assessment, policy development, and assurance functions. Equally important is the need for public health leadership to define clear success metrics (KPIs), create mechanisms for community feedback, and advocate for regulation that prioritizes public interests.

Component 6: Outcome-Focused Initiative: AI use in public health practice can be oriented toward improving population health outcomes. Every AI tool or resource adopted should be evaluated by its contribution to better health outcomes across the spectrum of prevention—primary, secondary, and tertiary. Products that fail to enhance quality of life, prevent diseases, promote health, protect well-being, thereby increase life expectancy, or foster active community engagement should be discontinued or replaced with more effective alternatives. This aligns with the Healthy People 2030 objectives, which emphasize equity, well-being, and measurable improvements in population health [109].

To ensure equitable outcomes, AI adoption in public health must explicitly address issues of access, equity, and inclusion. Digital interventions could be designed for rural and underserved populations, individuals with limited literacy, and people with disabilities, because without deliberate action, AI risks reinforcing the digital divide by privileging populations with existing infrastructure, connectivity, and technical literacy. Good AI interventions will dramatically expand access in regions with limited internet penetration, demonstrating how inclusive design can bridge structural inequities. By prioritizing health outcomes and equity, public health leaders can ensure that AI strengthens, rather than fragments, global health systems. This outcome-focused approach with equity-disaggregated evaluation directly addresses the ethical concerns about algorithmic bias and transparency raised in Section 5.

Considering these components, the following illustration demonstrates how the proposed framework can be applied to a complex and current public health challenge. Building on the AI applications described in Table 1, particularly risk stratification for prevention services and enhanced surveillance, consider a regional public health department seeking to deploy an AI-enabled tool to improve prevention, early identification, and care linkage for people with opioid use disorder (OUD), while addressing associated public health concerns including HIV, hepatitis C, mental health conditions, and infectious complications (Box 1). The illustrated integrated approach contrasts with typical practice, where AI tools are often adopted in isolation without community co-design, harm reduction principles, or explicit equity safeguards, increasing the risk of reinforcing existing disparities among already marginalized populations.

Box 1 **An example illustrating the application of the proposed Public Health AI Framework to opioid use disorder (OUD).**

**Figure 2:**
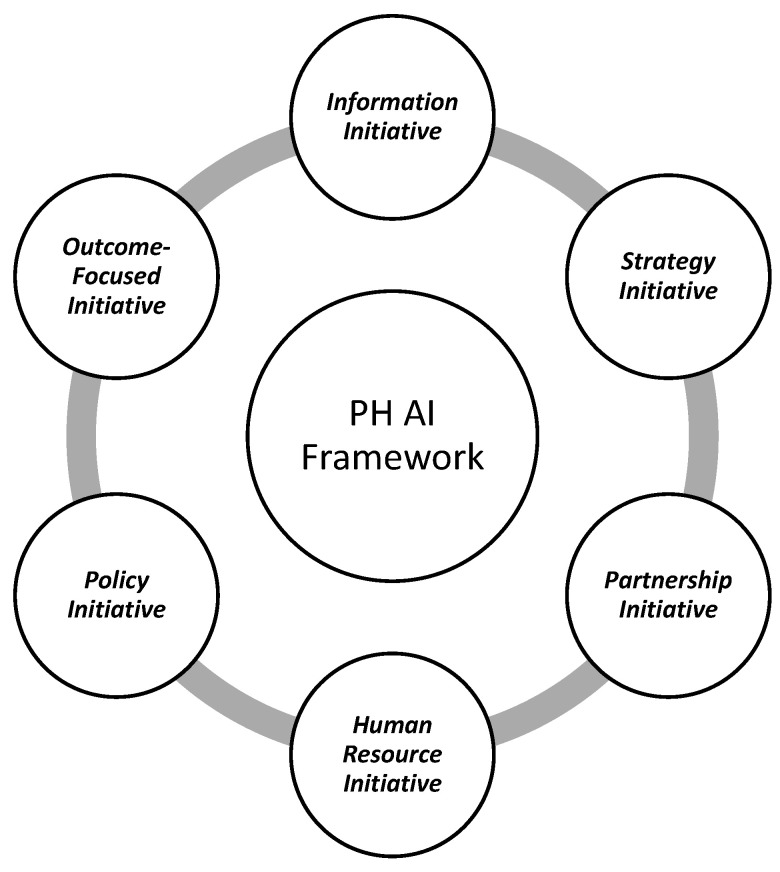
Public Health AI Framework.

Applying the proposed framework (Figure 2), leaders would first assess available surveillance data, overdose records, emergency department encounters, harm reduction program data, pharmacy dispensing records, and social determinants of health data to ensure representativeness across geographic areas, racial and ethnic groups, and risk populations (*Component 1: Information Initiative*). A clear strategic objective focused on strengthening engagement in OUD treatment, reducing fatal overdose events, and addressing co-occurring health risks would be defined alongside population-level and equity-focused indicators (*Component 2: Strategy Initiative*).

Partnerships would then be established with harm reduction organizations, addiction medicine specialists, infectious disease providers, mental health professionals, community pharmacists, data scientists, and individuals with lived experience to co-design risk stratification models, outreach strategies, and integrated care pathways (*Component 3: Partnership Initiative*). Workforce capacity would be strengthened through targeted training for epidemiologists, outreach workers, pharmacists, and case managers in algorithm interpretation, stigma reduction, harm reduction principles, and trauma-informed use of AI-generated insights (*Component 4: Human Resource Initiative*). Policy safeguards addressing privacy, informed consent, protection against criminalization, and appropriate data ownership and sharing across health and social services would be embedded prior to deployment (*Component 5: Policy Initiative*).

Finally, the system would be evaluated based on its impact on overdose prevention, engagement in OUD treatment, screening and linkage to care for HIV and hepatitis C, mental health outcomes, and equity across population subgroups, with iterative adjustments as needed (*Component 6: Outcome-Focused Initiative*).

Having presented the six components of the Public Health AI Framework and an example of its application, it is important to situate this approach within the broader landscape of AI governance and implementation models in health. Table 3 contrasts our framework with prevailing approaches across the six dimensions to clarify its distinctive conceptual contribution.

## 7. Discussion and Implications

**Figure 3:**
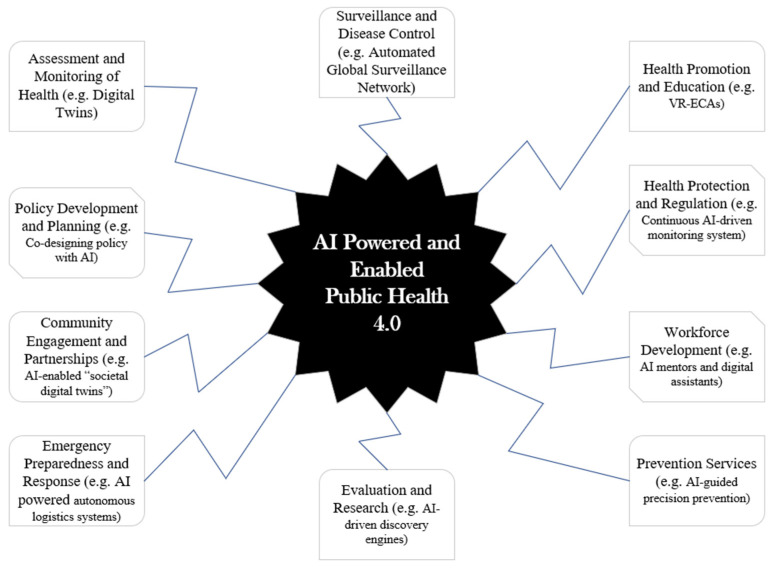
AI in public health practice.

Building on the conceptual framework and synthesis presented above, this section considers implications for how AI may shape public health practice under appropriate governance and oversight conditions. One of the core goals of Public Health 4.0 is AI-powered and -enabled public health systems (Figure 3).

Evidence shows that AI is currently redefining public health practice, impacting the 10 core functions of public health as shown above and in Table 1 and Table 2 [34,35,36,37,38,39,40,41,42,43,44,45,46,47,48,49]. However, strategic investment, capacity building, and inclusive innovation are immediately needed to ensure that no population is left behind. While AI systems today primarily support human decision-making, the field is evolving toward more integrated partnerships between AI and public health professionals. This will become more important in the long term when new and improved systems that anticipate, prevent, and respond to public health challenges at scale, such as Agentic AI, are fully operationalized. Also, AI should not just analyze data, but should work with experts to recommend policies, interventions, and strategies, as well as enabling precision public health that tailors interventions at the level of individuals and micro-communities, as recommended and outlined by the proposed framework.

Additionally, to maximize AI, public health professionals should remain cognitively engaged and serve as ethical stewards of the information produced by AI tools. With a focus on people, public health specialists are encouraged to insist on full implementation of this AI framework by developing appropriate AI adoption processes and procedures, ensuring that standard operating procedures are used to train public health practitioners and field workers to ensure proper AI use in the public health ecosystem.

AI in public health will be compromised if foundational challenges such as algorithmic bias, issues with transparency, data privacy, and exclusion of marginalized communities are not deliberately and proactively addressed. This is why public health leaders must work hand-in-glove with engineers, ethicists, policymakers, and community representatives to ensure that AI tools are trained on representative and inclusive datasets, outputs are explainable, and results are subject to continual evaluation and public scrutiny. To navigate an AI-enabled future, we should train a new generation of AI-literate public health professionals who understand not only the technical capabilities of AI but also its ethical, cultural, and operational implications. We could institute mentorship programs for emerging leaders to balance innovation with ethical stewardship, thus preparing them to lead cross-sector collaborations with engineers, data scientists, and policymakers. Public health institutions must embed ethical governance mechanisms into the lifecycle of AI implementation, including policies on informed consent, data ownership, community engagement, and grievance redress. Regulatory bodies and professional associations must continue to set and enforce standards for AI use in public health settings.

Failure to address current concerns may lead to existing disparities deepening and the erosion of public trust in digital health systems. Public health practitioners can ensure that human oversight remains a non-negotiable part of AI-assisted decisions, maximizing clear guidelines and training on ethical thresholds, incorporating appropriate use cases, and emphasizing the limitations of AI in complex or culturally sensitive situations. This is why it is a critical and timely strategic step to adopt the Public Health AI Framework as well as the ABC of AI-inclusive Decision-Making Process across all public health spaces to prevent misuse, misinformation, and inequity in evidence-based, context-specific research, and to align with population health goals for AI adoption [71].

The Public Health AI Framework has important implications for public health practice, education, and policy as the six components operate as an integrated governance mechanism rather than aspirational guidance. While the informative initiative ensures representative, high-quality data to mitigate bias and false signals; strategy initiative aligns AI use with public health functions, preventing ad hoc deployment; partnerships embed co-design, trust, and accountability across sectors; the human resources initiative addresses workforce readiness, ethics, and human-in-the-loop oversight; the policy initiative establishes guardrails for transparency, equity, and risk management; and the outcome-focused initiatives anchor AI deployment to measurable population health impact. Collectively, these components translate ethical intent into operational control, directly addressing failures related to governance gaps, workforce limitations, inequity, and poor implementation fidelity.

## 8. Conclusions

The AI evolution presents one of the most transformative opportunities in the history of public health. From personalized health interventions and multilingual communication to predictive surveillance and adaptive training, AI is already reshaping the foundations of public health practice. While engineers, data scientists, and entrepreneurs continue to push the boundaries of what AI *can do*, public health leaders must define what it *should do* in public health practice. The Public Health AI Framework positions public health professionals as co-designers and co-developers rather than passive adopters of AI technologies. By integrating data infrastructure, planning, partnership models, workforce development, policy safeguards, and outcome evaluation within a unified approach, the framework provides operational guidance. The framework has important practical implications for public health practice. It provides health departments with a roadmap for deliberate AI integration that embeds community co-design, harm reduction principles, and equity safeguards from the outset rather than as afterthoughts. For public health education, it signals the need to prepare professionals who can lead co-design processes. For policy, it underscores the necessity of sustained investment in data infrastructure, cross-sector partnerships, and governance mechanisms.

This framework is conceptual and has not been empirically validated through implementation studies; as such, its applicability may vary across jurisdictions with different resource capacities, governance structures, and technological infrastructure. These limitations point to important directions for future research, including testing the framework in diverse public health settings and identifying facilitators and barriers to adoption across different contexts.

## Figures and Tables

**Table 1:** Current applications of AI in public health.

Components	AI Applications	Outcomes
Assessment and Monitoring of Health	Advanced analytical techniques applied to derive actionable insights from EHRs, surveys, and social determinants to detect trends [34].	Faster identification of high-risk populations; development of real-time dashboards.
Surveillance and Disease Control	NLP/ML track social media, search queries, and news for early outbreaks [35,36,37]	Earlier outbreak detection, improved forecasting, and efficient tracing.
Health Promotion and Education	Chatbots and generative AI deliver personalized health education [38].	Increased engagement and improved health literacy.
Policy Development and Planning	Predictive models estimate health impacts of policies and strategies [39].	Data-driven policymaking, stronger cost–benefit, and more equitable planning.
Health Protection and Regulation	AI monitors food safety, environmental quality, and occupational health [40,41].	Proactive enforcement, reduced hazards, faster regulatory response.
Prevention Services	Risk stratification models guide preventive interventions and screenings [42].	Earlier diagnoses, cost-effective screening, and targeted efforts.
Workforce Development	AI-driven adaptive training platforms and simulations for the workforce [43,44].	Accessible training, reduced skill gaps, and better competency.
Community Engagement and Partnerships	Sentiment analysis and participatory platforms capture community needs [45].	Better alignment with community priorities, improved trust.
Emergency Preparedness and Response	AI-powered disaster modeling, resource allocation, and decision support [46,47].	Faster mobilization, optimized logistics, and reduced mortality in crises.
Evaluation and Research	AI accelerates reviews, data analysis, and simulation of interventions [48,49].	Stronger evidence synthesis, faster research, novel big data insights.

**Table 2:** Emerging AI applications in public health.

Components	Predicted Roles	Expected Outcomes
Assessment and Monitoring of Health	Use AI-powered “digital twins” of populations to simulate community health trends in real time [86].	To provide predictive analytics and preventive interventions that allow for early detection of health risks and proactive interventions in populations, not just retrospective analysis.
Surveillance and Disease Control	Automated global surveillance networks integrating health, climate, and travel data [87].	To detect outbreaks before the first local report.
Health Promotion and Education	Immersive AI-driven personalized health coaching (VR-ECAs) [88].	Can adapt to cultural context and personal motivation dynamically.
Policy Development and Planning	Co-designing policy with AI by stimulating economic, social, and health trade-offs instantly [89].	To predict long-term equity and economic impacts of policies in real time before implementation.
Health Protection and Regulation	Continuous AI-driven monitoring of supply chains, environmental systems, and workplaces [90,91].	Can flag, report, and even correct hazards in real time.
Prevention Services	AI-guided precision prevention at the individual genetic and behavioral level [92].	To deliver fully personalized preventive care recommendations integrated into daily life.
Workforce Development	AI mentors and digital assistants to guide public health professionals through tasks [93].	To provide context-aware coaching during fieldwork or emergencies.
Community Engagement and Partnerships	AI-enabled “societal digital twins” that model preemptive models in disease outbreaks and how proposed interventions will affect trust and equity [94,95].	To forecast community response to interventions before rollout, preventing mistrust and resistance.
Emergency Preparedness and Response	Fully autonomous logistics systems powered by AI and drones [96,97].	To support logistic systems in disasters and pre-position resources.
Evaluation and Research	AI-driven discovery engines that generate new hypotheses, design trials, and interpret results [98].	Conduct near real-time global meta-analyses and adapt interventions continuously as evidence evolves.

**Table 3:** Conceptual positioning of the Public Health AI Framework.

Description	Current AI Approaches in Healthcare	Public Health AI Framework
Primary purpose	Adoption of existing ethical governance or clinical implementation	Systematic co-creation and integration for population health
Target users	Policymakers, clinicians, and hospital administrators	Public health practitioners, epidemiologists, and health departments
Scope	Clinical care or cross-sectoral ethics principles	10 essential public health services and core public health functions
Role of public health	End-users of externally developed tools	Co-designers and co-developers across the AI lifecycle
Focus areas	Ethics, safety, clinical, and workflow integration	Integrated governance, workforce, data, partnerships, policy, and outcomes
Outcomes of interest	Ethical compliance or clinical effectiveness	Population health equity, prevention, and system resilience

## Data Availability

No new data were created or analyzed in this study.

## Abbreviations

The following abbreviations are used in this manuscript:

AI	Artificial Intelligence
CBT	Cognitive–Behavioral Therapy
CDC	U.S. Centers for Disease Control and Prevention
DL	Deep Learning
EHR	Electronic Health Records
EU	European Union
FDA	United States Food and Drug Administration
GenAI	Generative Artificial Intelligence
GIS	Geographic Information System
HIC	High-Income Countries
LMIC	Low- and Middle-Income Countries
MBO	Management By Objectives
ML	Machine Learning
NLP	Natural Language Processing
UNESCO	United Nations Educational, Scientific, and Cultural Organization
UNICEF	United Nations Children’s Fund
VR-ECAs	Virtual Reality-Embodied Conversational Agents
XAI	Explainable AI
WHO	World Health Organization
